# Nirmatrelvir and Molnupiravir and Post–COVID-19 Condition in Older Patients

**DOI:** 10.1001/jamainternmed.2023.5099

**Published:** 2023-10-23

**Authors:** Kin Wah Fung, Fitsum Baye, Seo H. Baik, Clement J. McDonald

**Affiliations:** 1 Lister Hill National Center for Biomedical Communications, National Library of Medicine, Bethesda, Maryland; 2 National Institutes of Health, Bethesda, Maryland

## Abstract

This observational cohort study assesses the occurrence of post–COVID-19 condition symptoms in Medicare enrollees prescribed nirmatrelvir and molnupiravir.

While the COVID-19 pandemic appears to be winding down, its effects are still felt by the millions of people worldwide experiencing post–COVID-19 condition (PCC, or long COVID).^1^ The antiviral drug nirmatrelvir (marketed as Paxlovid [Pfizer], in combination with ritonavir) and molnupiravir (Lagevrio [Merck]) are recommended as first- and second-line treatments for acute illness in patients with specific risk factors (eg, diabetes).^2^ However, there are still no US Food and Drug Administration–approved drugs for the treatment or prevention of PCC. Recent studies among US veterans (mostly male) suggest that nirmatrelvir and molnupiravir reduce the risk of some sequelae of COVID-19.^3,4^ We performed a cohort study of the 2 drugs in PCC in older patients who were Medicare enrollees.

## Methods

The cohort came from Medicare enrollees aged 65 years or older diagnosed with COVID-19 between January and September 2022. COVID-19 was identified with an outpatient *International Statistical Classification of Diseases, Tenth Revision, Clinical Modification* code of U07.1. In January 2022, free home COVID-19 tests became available and not all positive self-tests were captured in Medicare data. Therefore, we also considered the prescription of nirmatrelvir or molnupiravir to be indicative of COVID-19 because no other indications existed. Following previous work,^5^ we identified PCC based on the World Health Organization (WHO) consensus clinical definition.^6^ Any new occurrence (not present prior to COVID-19 diagnosis) of the 11 symptoms between 4 to 12 weeks after infection was considered as PCC. We used an extended Cox regression with propensity score adjustment to examine the 2 drugs and the incidence of PCC. We included age, sex, race, geographic region, dual eligibility, low-income subsidy, and 51 chronic comorbidities as covariates as included in the Medicare data (eMethods, eTable in Supplement 1). This study was declared not human participant research by the Office of Human Research Protection at the National Institutes of Health. Statistical analyses were conducted using SAS version 7.15 (SAS Institute Inc) and a 2-sided significance at *P* < .05. This study followed the STROBE reporting guideline.

## Results

Overall, among 3 975 690 outpatients with COVID-19, 57% remained in our study after exclusion. Among them, 19.5% received nirmatrelvir and 2.6% received molnupiravir. PCC incidence among patients receiving nirmatrelvir was 11.8%, 13.7% for molnupiravir, and 14.5% for neither, absolute risk reduction was 2.7% for nirmatrelvir, 0.8% for molnupiravir, with hazard ratios (HRs) of 0.87 (95% CI, 0.86-0.88; *P* < .001) for nirmatrelvir and 0.92 (95% CI, 0.90-0.94; *P* < .001) for molnupiravir, compared with no treatment (Table 1). Sensitivity analysis of only patients with the COVID-19 code showed a similar pattern but smaller effect sizes (nirmatrelvir: HR, 0.93 [95% CI, 0.92-0.94; *P* < .001], molnupiravir: HR, 0.96 [95% CI, 0.93-0.99; *P* = .001]). In an interaction analysis, we found significantly smaller effect sizes in females than males (HRs for nirmatrelvir: 0.89 vs 0.84; molnupiravir: 0.95 vs 0.88). Female sex; Asian, Black, and Hispanic races; and indicators of low income were associated with increased risk of PCC. The most common symptoms in PCC were fatigue (29.9%), dyspnea (22.4%), and cough (21%) (Table 2).

**Table 1.** Hazard Ratio Based on Cox Regression Model^a^

Index variable	Reference	No. (%)	Hazard ratio (95% CI)	Event rate, % (95% CI)		
				Index group	Reference group	Absolute risk reduction^b^
Nirmatrelvir	None	439 134 (19.5)	0.87 (0.86 to 0.88)	11.8 (11.7 to 11.9)	14.5 (14.4 to 14.6)	2.7
Molnupiravir	None	58 914 (2.6)	0.92 (0.90 to 0.94)	13.7 (13.5 to 14.0)	14.5 (14.4 to 14.6)	0.8
Female	Male	1 313 415 (58.5)	1.17 (1.16 to 1.18)	14.5 (14.4 to 14.6)	13.2 (13.1 to 13.2)	−1.3
Age, y						
70-74	65-69	656 324 (29.2)	0.78 (0.77 to 0.79)	12.7 (12.7 to 12.8)	12.0 (11.9 to 12.1)	−0.7
75-79	65-69	509 291 (22.7)	0.70 (0.69 to 0.71)	14.2 (14.1 to 14.3)	12.0 (11.9 to 12.1)	−2.2
80-84	65-69	324 008 (14.4)	0.64 (0.63 to 0.66)	15.8 (15.7 to 16.0)	12.0 (11.9 to 12.1)	−3.8
≥85	65-69	313 754 (14.0)	0.61 (0.60 to 0.63)	16.9 (16.7 to 17.0)	12.0 (11.9 to 12.1)	−4.9
Race^c^						
Asian	White	81 073 (3.6)	1.10 (1.07 to 1.12)	13.3 (13.0 to 13.5)	13.9 (13.9 to 14.0)	0.6
Black	White	82 249 (3.7)	1.24 (1.22 to 1.27)	15.3 (15.0 to 15.5)	13.9 (13.9 to 14.0)	−1.4
Hispanic	White	93 325 (4.2)	1.02 (1.00 to 1.04)	15.4 (15.1 to 15.6)	13.9 (13.9 to 14.0)	−1.5
Other^d^	White	93 011 (4.1)	1.04 (1.02 to 1.06)	12.4 (12.1 to 12.6)	13.9 (13.9 to 14.0)	1.5
Income						
Dual eligibility	Nondual	244 874 (10.9)	1.06 (1.05 to 1.08)	16.6 (16.5 to 16.8)	13.6 (13.5 to 13.6)	−3.0
Low-income subsidy	Nondual	21 049 (0.9)	1.07 (1.03 to 1.10)	16.4 (15.9 to 16.9)	13.6 (13.5 to 13.6)	−2.8
Region						
Midwest	Northeast	443 777 (19.8)	0.91 (0.90 to 0.92)	13.3 (13.2 to 13.4)	14.1 (14.1 to 14.2)	0.8
South	Northeast	869 144 (38.7)	0.95 (0.94 to 0.97)	14.2 (14.2 to 14.3)	14.1 (14.1 to 14.2)	−0.1
West	Northeast	423 314 (18.8)	1.04 (1.03 to 1.06)	13.7 (13.6 to 13.8)	14.1 (14.1 to 14.2)	0.4
Other	Northeast	16 706 (0.7)	0.52 (0.49 to 0.56)	14.0 (13.5 to 14.5)	14.1 (14.1 to 14.2)	0.1

^a^ The 51 chronic comorbidities that are included as covariates are not shown in this table.

^b^ Absolute risk reduction is the difference of raw event rate between reference and index groups (reference – index). It is possible that the directionality of absolute risk reduction can be different from that indicated by the hazard ratio adjusted for covariates and propensity scores.

^c^ Race categories are included as found in Medicare data and are included as potential factors in the outcomes of COVID-19.

^d^ The race classification aligns with that in the US Centers for Medicare & Medicaid Services Virtual research data Center database, which was American Indian or Alaska Native, Asian, Black, Hispanic, White, other, and unknown. We combined American Indian or Alaska Native, other, and unknown into other because of the small numbers in these categories.

**Table 2.** Characteristics of Patients With Post–COVID-19 Condition

Characteristic	No. of patients (%)				Standardized mean difference	
	Overall (N = 313 262)	Nirmatrelvir (n = 51 658)	Molnupiravir (n = 8089)	None (n = 253 617)	Nirmatrelvir vs none	Molnupiravir vs none
Post–COVID-19 condition symptom						
Fatigue/malaise/weakness	93 653 (29.9)	15 049 (29.1)	2292 (28.3)	76 338 (30.1)	−0.02	−0.04
Dyspnea	70 306 (22.4)	10 810 (20.9)	1811 (22.4)	57 707 (22.8)	−0.04	−0.01
Cough	65 660 (21.0)	10 910 (21.1)	1737 (21.5)	53 035 (20.9)	0.01	0.01
Chest pain	55 506 (17.7)	8905 (17.2)	1432 (17.7)	45 185 (17.8)	−0.02	0.00
Palpitations	37 734 (12.0)	5943 (11.5)	919 (11.4)	30 887 (12.2)	−0.02	−0.03
Headache	25 704 (8.2)	3983 (7.7)	701 (8.7)	21 033 (8.3)	−0.02	0.01
Muscle/joint pain	23 174 (7.4)	4205 (8.1)	605 (7.5)	18 368 (7.2)	0.03	0.01
Memory problem	13 093 (4.2)	2194 (4.2)	350 (4.3)	10 552 (4.2)	0.00	0.01
Cognitive impairment	8096 (2.6)	1070 (2.1)	190 (2.3)	6837 (2.7)	−0.04	−0.02
Sleep disturbance	3844 (1.2)	699 (1.4)	86 (1.1)	3059 (1.2)	0.01	−0.01
Loss of taste/smell	1321 (0.4)	220 (0.4)	30 (0.4)	1071 (0.4)	0.00	−0.01
Female	190 372 (60.8)	31 176 (60.4)	4684 (57.9)	154 571 (60.9)	−0.01	−0.06
Age range, y						
65-69	53 348 (17.0)	10 264 (19.9)	1226 (15.2)	41 873 (16.5)	0.09	−0.04
70-74	83 569 (26.7)	15 800 (30.6)	2119 (26.2)	65 674 (25.9)	0.11	0.01
75-79	72 104 (23.0)	12 166 (23.6)	1985 (24.5)	57 978 (22.9)	0.02	0.04
80-84	51 308 (16.4)	7581 (14.7)	1436 (17.8)	42 317 (16.7)	−0.05	0.03
≥85	52 933 (16.9)	5847 (11.3)	1323 (16.4)	45 775 (18.0)	−0.18	−0.04
Race^a^						
Asian	10 762 (3.4)	2017 (3.9)	145 (1.8)	8607 (3.4)	0.03	−0.09
Black	12 549 (4.0)	1297 (2.5)	227 (2.8)	11 026 (4.3)	−0.09	−0.07
Hispanic	14 327 (4.6)	1719 (3.3)	284 (3.5)	12 328 (4.9)	−0.07	−0.06
White	264 131 (84.3)	44 414 (86.0)	7172 (88.7)	212 630 (83.8)	0.06	0.13
Other^b^	11 493 (3.7)	2211 (4.3)	261 (3.2)	9026 (3.6)	0.04	−0.02
Income						
Dual eligibility	40 725 (13.0)	3206 (6.2)	638 (7.9)	36 892 (14.5)	−0.25	−0.19
Nondual, low-income subsidy	3454 (1.1)	378 (0.7)	82 (1.0)	2994 (1.2)	−0.04	−0.01
Nondual, no low-income subsidy	269 083 (85.9)	48 074 (93.1)	7369 (91.1)	213 731 (84.3)	0.25	0.19
Region						
Northeast	69 890 (22.3)	11 205 (21.7)	1059 (13.1)	57 639 (22.7)	−0.02	−0.23
Midwest	59 226 (18.9)	9924 (19.2)	1569 (19.4)	47 749 (18.8)	0.01	0.02
South	123 707 (39.5)	19 899 (38.5)	4444 (54.9)	99 416 (39.2)	−0.01	0.32
West	58 099 (18.5)	10 370 (20.1)	949 (11.7)	46 801 (18.5)	0.04	−0.18
Other	2340 (0.7)	260 (0.5)	68 (0.8)	2012 (0.8)	−0.03	0.01

^a^ Race categories are included as found in Medicare data and are included as potential factors in the outcomes of COVID-19.

^b^ The race classification aligns with that in the US Centers for Medicare & Medicaid Services Virtual research data Center database, which was American Indian or Alaska Native, Asian, Black, Hispanic, White, other, and unknown. We combined American Indian or Alaska Native, other, and unknown into other because of the small numbers in these categories.

## Discussion

Consistent with the findings of Xie et al,^3,4^ we found that nirmatrelvir and molnupiravir were associated with a small reduction in incidence of PCC. Our effect sizes are smaller than those of Xie et al^3,4^ (absolute risk reduction, nirmatrelvir 4.5%; molnupiravir 3.0%) but our sample size is 8-fold larger. We also have a more balanced sex ratio (female 59% vs 14%), which is important because PCC is more common in females. The smaller effect sizes in females may explain our overall smaller effect sizes. We used the WHO consensus definition based on symptoms rather than disease diagnosis (eg, ischemic heart disease), which is more akin to how PCC is identified clinically. Limitations of our study include not incorporating vaccination status because of incomplete data, use of prescription of the drugs as evidence of COVID-19, and restriction to patients 65 years or older. The current approved use of the 2 drugs is for the prevention of severe acute COVID-19. Our findings suggest that they may also have a role in preventing PCC.

## Notes

Supplement 1.
eMethods

eReferences
**eTable 1.** List of 51 Comorbidities

Supplement 2.
Data Sharing Statement
