# Supplementary material for: Nirmatrelvir and Molnupiravir and Post–COVID-19 Condition in Older Patients
Source: JAMA Intern Med. 2023 Oct 23;183(12):1404–6. doi: 10.1001/jamainternmed.2023.5099 (PMC10594174; doi:10.1001/jamainternmed.2023.5099)
Supplement: Supplement 1. — eMethods eReferences eTable 1. List of 51 Comorbidities [file jamainternmed-e235099-s001.pdf]

Supplemental Online Content

Fung KW, Baye F, Baik, SH, McDonald CJ. Nirmatrelvir and molnupiravir associated with reduced risk of long COVID in elderly patients. *JAMA Intern Med.* Published online October 23, 2023. 10.1001/jamainternmed.2023.5099

eMethods.

eReferences.

eTable 1. List of 51 comorbidities

This supplemental material has been provided by the authors to give readers additional information about their work.

© 2023 American Medical Association. All rights reserved.

eMethods

1. Study population

The primary cohort of this study was all Medicare patients aged 65 and ever diagnosed to have COVID-19 between January and September 2022. Through the Virtual Research Data Center (VRDC) of the Centers for Medicare and Medicaid Services (CMS), [1] we accessed de-identified encounter and medication data of all Medicare beneficiaries in 2021 and 2022. We focused our analysis on Medicare beneficiaries aged ≥65, since younger Medicare beneficiaries were not representative of the general population aged <65 as they needed qualifying disability conditions to enroll. We identified outpatients with COVID-19 by the ICD-10-CM code U07.1 assigned in an outpatient encounter, who were not admitted to an inpatient facility for COVID-19 within 4 weeks of COVID-19 diagnosis. We focused on outpatients because medication information for inpatients may not be complete in outpatient prescription drug claim data (Part D). Since rapid antigen self-tests for COVID-19 were available and since January 2022, they were made free at the request of all people, including Medicare enrollees, some patients with a positive self-test may not report the diagnosis to their providers. Therefore, we also considered the prescription of nirmatrelvir or molnupiravir to be indicative of COVID-19 because there were no other indications for these drugs.

To ensure that we have sufficient data for the one year look-back period for long COVID symptoms (see below for method to identify long COVID), we excluded patients a) with less than one year of Medicare coverage, b) with no encounters in a year prior to COVID-19, c) who were continuously enrolled in Medicare Advantage plans in the period between 1 year before and 12 weeks after the COVID-19 diagnosis. The last exclusion is necessary because encounter data for patients enrolled in non-fee-for-service plans were not yet available during our study period. This study was declared not human subject

© 2023 American Medical Association. All rights reserved.

research by the Office of Human Research Protection at the National Institutes of Health and by the CMS's Privacy Board.

2. Identifying long COVID

For identification of long COVID, we followed the method of our previous study [2] and used the World Health Organization's (WHO) consensus clinical definition,[3] which was developed through a consensus process involving over 200 experts, researchers and patients:

> *"Post COVID-19 condition occurs in individuals with a history of probable or confirmed SARS-CoV-2 infection, usually 3 months from the onset of COVID-19 with symptoms that last for at least 2 months and cannot be explained by an alternative diagnosis. Common symptoms include fatigue, shortness of breath, cognitive dysfunction but also others which generally have an impact on everyday functioning. Symptoms may be new onset, following initial recovery from an acute COVID-19 episode, or persist from the initial illness. Symptoms may also fluctuate or relapse over time."*

We used ICD-10-CM codes to identify the 11 symptoms that at least 50% of the participants in the WHO's consensus building process thought were critical to include. Long COVID was defined as presence of any of the 11 symptoms unless they were excluded (see below for exclusion criteria). For the onset of symptoms, we followed the guidelines for identification of long COVID developed by the U.S. Centers for Disease Control and Prevention [a] (CDC)[4] and the United Kingdom's National Institute

---

[a] Centers for Disease Control and Prevention uses "Post-COVID19 conditions" as an umbrella term for the wide range of physical and mental health consequences experienced by some patients that are present four or more weeks after SARS-CoV-2 infection, including by patients who had initial mild or asymptomatic acute infection; equivalent to the lay term, "Long COVID"

© 2023 American Medical Association. All rights reserved.

for Health and Care Excellence [b] (NICE),[5] and looked for long COVID symptoms from 4 to 12 weeks after the COVID-19 diagnosis. For patients without a COVID-19 diagnosis code, the prescription date of nirmatrelvir or molnupiravir was used as the diagnosis date. To satisfy the requirement that “[the symptom] cannot be explained by an alternative diagnosis”, we excluded a long COVID symptom if it was reported in that patient from 2 weeks to 1 year before the COVID-19 diagnosis. We started the look-back period 2 weeks prior to COVID-19 diagnosis because COVID-19-related symptoms started to increase from 2 weeks before the COVID-19 diagnosis, indicating a lag in diagnosis reporting.

We used the symptom-based method to identify long COVID, instead of relying on the specific ICD-10-CM code (U09.9 Post COVID-19 condition), because our previous study[2] showed that using the code-based method would result in significant under-reporting.

3. Drug exposure, comorbidities and other factors

We identified the two study drugs by matching their generic names in the Medicare outpatient prescription drug (Part D) data. For patients who were identified using the COVID-19 diagnosis code, the prescription date had to be within ±5 days of the COVID-19 diagnosis. For patients with multiple episodes of COVID-19 within the study period, we only counted the first episode. We included 51 comorbidities (chronic conditions) with >1% prevalence in our study population that were tracked by CMS in the Chronic Conditions Data Warehouse.[6] We used demographics and socioeconomic indicators (age, sex, race, geographic region, dual eligibility, low-income subsidy) as included in the Medicare data. We decided against including COVID-19 vaccine status because of incomplete data.

[b] National Institute for Health and Care Excellence guideline defines long covid as “signs and symptoms that develop during or following an infection consistent with covid-19 and which continue for more than four weeks and are not explained by an alternative diagnosis.”

© 2023 American Medical Association. All rights reserved.

This is likely related to the wide availability of free COVID-19 vaccination outside the traditional healthcare providers (e.g., public health clinics, community pharmacies). Based on Medicare data, only 38% of our study population had received at least one dose of COVID-19 vaccine. However, according to CDC statistics, 95% of the population ≥ 65 had received at least one dose.[7] Since almost all study individuals were vaccinated, it would not be necessary to adjust for the vaccine status.

4. Statistical analysis

The primary outcome was long COVID, as defined above. We followed all patients starting from the day of COVID-19 diagnosis, or prescription day of the study drugs if there was no COVID-19 code, until long COVID, death, disenrollment from Medicare Parts A/B/D or 12 weeks after COVID-19 diagnosis, whichever came first. In order to mitigate selection bias with the 3 exposure groups (nirmatrelvir, molnupiravir and no treatment), we developed propensity scores (PSs) separately for each study drug using logistic regressions. The PS was the likelihood of receiving a study drug, conditional on patient's age, sex, race, geographic region, dual eligibility, low-income subsidy, and 51 chronic comorbidities at the baseline. To ensure that the 3 exposure groups were balanced, we used the doubly robust method by running the Cox regression with PSs as additional adjustments, and including the same patient characteristics in PS calculation as covariates. We did a sensitivity analysis restricted to patients identified only with the COVID-19 diagnosis code. We also did an interaction analysis with two interaction terms (female*nirmatrelvir and female*molnupiravir) to see whether the effects were the same among males and females.

This study is reported as per the Strengthening the Reporting of Observational Studies in Epidemiology (STROBE) guideline.

© 2023 American Medical Association. All rights reserved.

eReferences

1. Research Data Assistance Center (ResDAC). CMS Virtual Research Data Center (VRDC). https://www.resdac.org/cms-virtual-research-data-center-vrdc
2. Fung KW, Baye F, Baik SH, Zheng Z, McDonald CJ. Prevalence and characteristics of long COVID in elderly patients: An observational cohort study of over 2 million adults in the US. *PLoS medicine*. Apr 17 2023;20(4):e1004194. doi:10.1371/journal.pmed.1004194
3. World Health Organization. A clinical case definition of post COVID-19 condition by a Delphi consensus. http://www.fx1234.com/iris/bitstream/handle/10665/345824/WHO-2019-nCoV-Post-COVID-19-condition-Clinical-case-definition-2021.1-eng.pdf?sequence=1&isAllowed=y
4. US Department of Health & Human Services. National Research Action Plan on Long COVID. https://www.covid.gov/assets/files/National-Research-Action-Plan-on-Long-COVID-08012022.pdf
5. Sivan M, Taylor S. NICE guideline on long covid. *BMJ*. Dec 23 2020;371:m4938. doi:10.1136/bmj.m4938
6. Centers for Medicare & Medicaid Services. Chronic Conditions Data Warehouse: CCW Chronic Condition Algorithms. . https://www.ccwdata.org/web/guest/condition-categories
7. Centers for Disease Control and Prevention. COVID-19 Vaccinations in the United States. https://covid.cdc.gov/covid-data-tracker/#vaccinations_vacc-people-booster-percent-pop5

© 2023 American Medical Association. All rights reserved.

eTable1 List of 51 comorbidities

Acute myocardial infarction
Atrial Fibrillation
Cataract
Chronic Kidney Disease
Chronic obstructive pulmonary disease
Heart Failure
Diabetes
Glaucoma
Hip/Pelvic Fracture
Ischemic Heart Disease
Depression
Alzheimer's Disease Or Dementia
Osteoporosis
Rheumatoid Arthritis/Osteoarthritis
Stroke/Transient Ischemic Attack
Breast Cancer
Colorectal Cancer
Prostate Cancer
Lung Cancer
Endometrial Cancer
Anemia
Asthma
Hyperlipidemia
Hyperplasia
Hypertension
Hypothyroidism
Attention deficit hyperactivity disorder And Other Conduct Disorders
Alcohol Use Disorders
Anxiety Disorders
Bipolar Disorder
Traumatic Brain Injury
Drug Use Disorder
Personality Disorders
Schizophrenia
Post-Traumatic Stress Disorder
Epilepsy
Cystic Fibrosis
Fibromyalgia, Chronic Pain And Fatigue
Viral Hepatitis (General)
Liver Disease Cirrhosis
Leukemias And Lymphomas
Migraine And Other Chronic Headache
Mobility Impairments
Obesity
Overarching Opioid Use Disorder
Peripheral Vascular Disease
Spinal Cord Injury
Tobacco Use Disorders
Pressure Ulcers And Chronic Ulcers
Deafness And Hearing Impairment
Blindness And Visual Impairment

© 2023 American Medical Association. All rights reserved.
