# Supplementary material for: Nirmatrelvir and Molnupiravir and Post–COVID-19 Condition in Older Patients
Source: JAMA Intern Med. 2023 Oct 23;183(12):1404–6. doi: 10.1001/jamainternmed.2023.5099 (PMC10594174; doi:10.1001/jamainternmed.2023.5099)
Supplement: Supplement 2. — Data Sharing Statement [file jamainternmed-e235099-s002.pdf]

Data Sharing Statement

Fung. Nirmatrelvir and Molnupiravir and Long COVID in Older Patients. *JAMA Intern Med*. Published October 23, 2023. doi:10.1001/jamainternmed.2023.5099

Data
Data available: No

Additional Information
Explanation for why data not available: Concerning data availability, CMS does not let us download (or distribute) any patient level data. The data stay on their machine, and we analyze it with software they provide on their machine. If researchers wish to access the raw data, they can contact the CMS Virtual Research Data Center https://resdac.org/cms-virtual-research-data-center-vrdc. However, data access requires the payment of a fee.
